# Impaired tissue perfusion in high-risk patients having major abdominal surgery: a multicenter observational study

**DOI:** 10.1186/s13054-026-05940-y

**Published:** 2026-03-11

**Authors:** Moritz Flick, Sebastian Schmid, Fabian Müller-Graf, Amelie Zitzmann, Bettina Jungwirth, Kristina Fuest, Catherina Bubb, Maria Fischer, Thorsten Annecke, Lilay Kidane, Dominik Jarczak, Linda Krause, Sebastian Rehberg, Bernd Saugel, Daniel A. Reuter, German Society of Anaesthesiology Intensive Care (GSAIC) Trials Group, Alexander Fuchs, Dominik Wrede, Lena Bork, Raimung Hof, Axel Nierhaus, Karim Kouz, Sarah S Grotheer, Sophie Perchermaier, Anna Scholze

**Affiliations:** 1 Department of Anesthesiology, Center of Anesthesiology and Intensive Care Medicine, University Medical Center Hamburg-Eppendorf, Martinistrasse 52, 20246 Hamburg, Germany; 2 Department of Anaesthesiology and Intensive Care Medicine, University Hospital Ulm, Ulm, Germany; 3 Department of Anesthesiology, Intensive Care Medicine and Pain Therapy, University Medical Center Rostock, Rostock, Germany; 4 Department of Anaesthesiology and Intensive Care Medicine, TUM University Hospital Rechts der Isar, Munich, Germany; 5 Department of Anesthesiology and Intensive Care Medicine, Faculty of Medicine, University Hospital of Cologne, University of Cologne, Cologne, Germany; 6 Department of Anesthesiology and Intensive Care Medicine, Cologne Merheim Medical Center, University of Witten/Herdecke, Cologne, Germany; 7 Department of Intensive Care Medicine, University Medical Center Hamburg-Eppendorf, Hamburg, Germany; 8 Institute of Medical Biometry and Epidemiology, University Medical Center Hamburg-Eppendorf, Hamburg, Germany; 9 Department of Anaesthesiology, Intensive Care, Emergency Medicine, Transfusion Medicine and Pain Therapy, Medical School and University Medical Center OWL, Campus Bielefeld-Bethel, Bielefeld, Germany; 10 Outcomes Research Consortium®, Houston, TX USA

**Keywords:** Anesthesia, Microcirculation, Tissue perfusion, Capillary refill time, Mottling, Lactate, Postoperative care

## Abstract

**Background:**

Surgical trauma may cause systemic inflammation and impaired tissue perfusion. However, the incidence of signs of impaired tissue perfusion in patients undergoing major abdominal surgery remains largely unknown. This study investigated the incidence of signs of impaired tissue perfusion – specifically capillary refill time, mottling score, lactate, and central venous oxygen saturation – in high-risk patients having major abdominal surgery.

**Methods:**

This multicenter prospective observational study included 291 high-risk patients undergoing major abdominal surgery at 5 university hospitals in Germany. Signs of impaired tissue perfusion were measured until the first postoperative day and defined as a capillary refill time of ≥ 3 s, mottling score of ≥ 1, lactate of ≥ 2 mmol/L, or central venous oxygen saturation of ≤ 65%. The primary endpoint was the proportion of patients with signs of impaired tissue perfusion until the first postoperative day.

**Results:**

Overall, 171 patients (59%) demonstrated signs of impaired tissue perfusion at any postoperative time point. Specifically, capillary refill time was prolonged in 19 patients (7%), mottling score was elevated in 43 patients (15%), lactate levels were elevated in 73 patients (25%), and central venous oxygen saturation was low in 119 patients (41%). At each time point, the majority of patients exhibited only a single sign of impaired tissue perfusion. No meaningful differences in macrocirculation variables were observed between patients with and without signs of impaired tissue perfusion. The presence of any postoperative sign of impaired tissue perfusion was weakly associated with longer intensive care unit length of stay (*P* = 0.012), but not meaningfully with the highest SOFA score on postoperative days 1, 3, or 5, hospital length of stay, and 28-day mortality.

**Conclusions:**

Approximately 60% of high-risk patients undergoing major abdominal surgery demonstrated postoperative signs of impaired tissue perfusion, although most patients exhibited only one sign. Whether these signs of impaired tissue perfusion are clinically meaningful requires further investigation in larger studies.

**Trial Registration:**

German Clinical Trials Register (DRKS00020264) on January 10, 2020.

**Supplementary Information:**

The online version contains supplementary material available at 10.1186/s13054-026-05940-y.

## Introduction

Impairment of the microcirculation is a hallmark of sepsis and is associated with organ injury and mortality [1, 2]. During major abdominal surgery, surgical trauma may cause systemic inflammation [3, 4]. Therefore, it is often assumed that patients having major abdominal surgery are also at risk of developing impaired tissue perfusion [5]. In clinical practice, however, the microcirculation usually is not systematically assessed and postoperative tissue perfusion, therefore, is scarcely investigated.

The microcirculation is a complex system that cannot be assessed with a single measurement [5, 6]. Therefore, several non-invasive or minimally invasive markers have been proposed for assessing different aspects of tissue perfusion. These include clinical markers such as capillary refill time [7–10] and skin mottling score [11], as well as laboratory markers such as arterial lactate levels and central venous oxygen saturation [12]. However, despite the well-recognized importance of tissue perfusion and the ability to assess it at the bedside, it remains largely unknown how commonly patients having major abdominal surgery develop signs of impaired tissue perfusion.

We, therefore, performed a multicenter observational study to investigate the incidence of signs of impaired tissue perfusion – specifically capillary refill time, mottling score, lactate, and central venous oxygen saturation – in high-risk patients having major abdominal surgery. We additionally investigated the relationship between signs of impaired tissue perfusion and the maximum postoperative sequential organ failure assessment (SOFA) score, intensive care unit length of stay, hospital length of stay, and 28-day mortality.

## Materials and methods

### Study design

This prospective multicenter observational study was performed in five university medical centers in Germany (Hamburg, Cologne, Rostock, Ulm, and Munich [TUM University Hospital]) between April 2019 and October 2021 in accordance with the principles of the Declaration of Helsinki. The study was primarily approved by the ethics committee of the University Hospital Greifswald, Germany (BB 035/18; September 12, 2018) and subsequently approved by the ethics committees of all participating hospitals. This study was retrospectively registered at the German Clinical Trials Register (DRKS00020264) on January 10, 2020. The statistical analysis plan was approved by the principal investigators and study statistician before the analysis. The study is reported according to the STROBE (STrengthening the Reporting of OBservational studies in Epidemiology) statement [13].

### Patients

We enrolled patients ≥ 18 years scheduled for elective major abdominal surgery (general, urology, gynecology) expected to last at least 120 min with clinical indication for an arterial and central venous catheter who were expected to be transferred to an intensive care unit after surgery. We excluded patients with peripheral artery occlusive disease Fontaine IIb or higher, patients with vasculitis, and patients unable to provide informed consent. We also excluded pregnant women.

### Protocol

All patients were treated according to routine care without any specific protocol. We assessed the following signs of impaired tissue perfusion after induction of general anesthesia before the start of surgery, 2 h after surgery, 6 h after surgery, and on the first postoperative day: capillary refill time, mottling score, arterial lactate, and central venous oxygen saturation. Capillary refill time and mottling score were assessed by study personnel or anesthesiologists working in the intensive care unit.

The capillary refill time was assessed manually by applying firm pressure to a distal capillary bed typically at the distal phalanx of the index finger for 5 s [8]. Upon complete release of pressure, the time to return of baseline skin color was measured in seconds using a stopwatch. Timing was initiated at complete release of pressure and stopped at visual return of baseline skin color. Measurements were performed once per assessment. The mottling score was assessed based on the anatomical extent of mottling anterior to the knee [11]. Specifically, a score of 0 denotes the absence of mottling; a score of 1 refers to a small, coin-sized area of mottling confined to the center of the knee; a score of 2 describes mottling extending to, but not beyond, the upper border of the patella; a score of 3 indicates spread to the mid-thigh; a score of 4 to the groin fold; and a score of 5 reflects extensive mottling beyond the groin. Arterial lactate and central venous oxygen saturation were determined using point-of-care blood gas analysis. We considered a patient having ‘signs of impaired tissue perfusion’ when the capillary refill time was ≥ 3 s, the mottling score was ≥ 1, lactate was ≥ 2 mmol/L, or central venous oxygen saturation was ≤ 65%.

To evaluate organ injury, we calculated the SOFA score on postoperative days 1, 3, and 5. For all assessed variables, missing values were considered as normal. We also documented postoperative intensive care unit length of stay, hospital length of stay, and 28-day mortality. Patient characteristics and perioperative data were extracted from the patients’ electronic medical file.

### Endpoints

The primary endpoint was the proportion of patients with signs of impaired tissue perfusion after surgery and until the first postoperative day. Secondary endpoints were the individual proportions of patients who developed abnormal capillary refill time, mottling score, lactate, and central venous oxygen saturation (each marker individually). Additional endpoints were the proportion of patients with impaired tissue perfusion (any vs. none and each marker individually) at each measurement point. Finally, we analyzed the correlation between signs of impaired tissue perfusion (any sign and each sign individually) with the single maximum SOFA score on postoperative days 1, 3, or 5, intensive care unit length of stay, hospital length of stay, and 28-day mortality. On an exploratory level, we assessed the correlation of risk factors (i.e., patient characteristics or perioperative data) and signs of impaired tissue perfusion.

### Statistical analysis

Numeric variables are presented as median and 25th and 75th percentile. Categorical variables are presented as numbers and percentages. The primary and secondary endpoints were analyzed descriptively using absolute numbers and percentages. We performed a predefined sensitivity analysis restricted to patients who developed signs of ‘new-onset impaired tissue perfusion’, i.e., patients who did not have signs of impaired tissue perfusion before surgery, but developed signs of impaired tissue perfusion after surgery. The association between signs of impaired tissue perfusion (any sign and each sign individually) with the single maximum SOFA score on postoperative days 1, 3, or 5, intensive care unit length of stay, hospital length of stay, and 28-day mortality was analyzed using two-sample Wilcoxon tests. The correlation between the highest postoperative lactate, mottling score, capillary refill time, and lowest central venous oxygen saturation compared to maximum postoperative SOFA score on days 1, 3 or 5, intensive care unit length of stay, and hospital length of stay was analyzed using Spearman’s rank correlation coefficients with 95% confidence intervals computed by 1000 bootstrap replicates. The association between 28-day mortality and the highest postoperative lactate, mottling score, capillary refill time, and lowest central venous oxygen saturation was analyzed using a two-sample t-test. The association of risk factors (i.e., patient characteristics or perioperative data) and signs of impaired tissue perfusion was analyzed using two-sample Wilcoxon tests.

Due to limited available data, no formal sample size calculation was performed. Based on the simple asymptotic formula for the confidence interval for one proportion, the sample size of 291 patients creates a 95%-confidence interval with a width of 11.3% around the observed incidence of 59%.

## Results

We included 291 patients in this study. All included patients were analyzed. Patient characteristics and perioperative data are presented in Table 1.

**Table 1:** Patient Characteristics

Variable		*n* = 291
Site, n	Hamburg	100 (34%)
	Cologne	56 (19%)
	Munich	52 (18%)
	Ulm	50 (17%)
	Rostock	33 (11%)
Age, years		64 (56 to 72)
Female, n		158 (54%)
ASA physical status class I/II/III/IV, n		10/110/165/6
Epidural catheter, n		233 (81%)
Type of surgery		
	General oncologic, n	12 (4%)
	Upper gastrointestinal, n	46 (16%)
	Lower gastrointestinal, n	13 (4%)
	Pancreatic, n	70 (24%)
	Hepatobiliary, n	53 (18%)
	Urological, n	19 (7%)
	Gynecological, n	72 (25%)
	Other, n	6 (2%)
Medical History		
	Arterial Hypertension, n	113 (39%)
	Coronary artery disease, n	35 (12%)
	Diabetes mellitus, n	49 (17%)
	Chronic kidney disease, n	24 (8%)
	Chronic obstructive pulmonary disease, n	13 (5%)
Duration of surgery, min		285 (199 to 360)
Blood loss, ml		400 (100 to 800)
Intraoperative crystalloids, ml		3500 (2131 to 4868)
Intraoperative colloids, ml		480 (0 to 1000)
Maximum postoperative SOFA score, au		3 (1 to 4)
	Maximum SOFA score on postoperative day 1, n	275 (95%)
	Maximum SOFA score on postoperative day 3, n	13 (5%)
	Maximum SOFA score on postoperative day 5, n	3 (1%)
Intensive care unit length of stay, days		2 (2 to 4)
Hospital length of stay, days		13 (9 to 20)
28-day mortality*, n		10 (3%)

Data are presented as number (percentage) or median (25^th^ to 75^th^ percentile). *ASA - American Society of Anesthesiologists; SOFA - Sequential Organ Failure Assessment*. *Due to loss to follow-up, 28-day mortality was assessed in only 287 patients

**Fig. 1:**
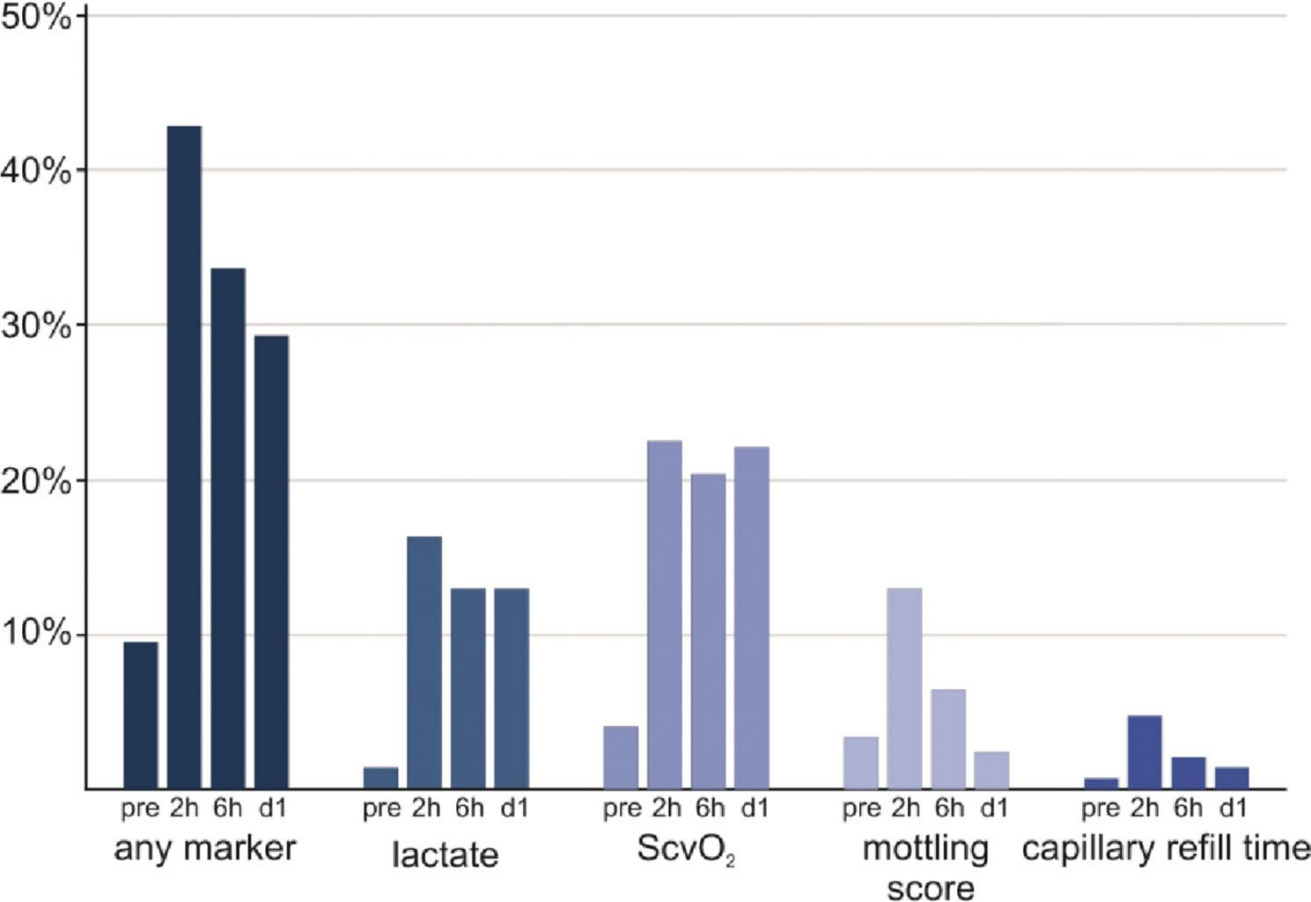
Individual signs of impaired tissue perfusion. Bar chart illustrating the number of patients having each sign of impaired tissue perfusion per time point. *ScvO2 – central venous oxygen saturation; D1 – postoperative day 1*

**Fig. 2:**
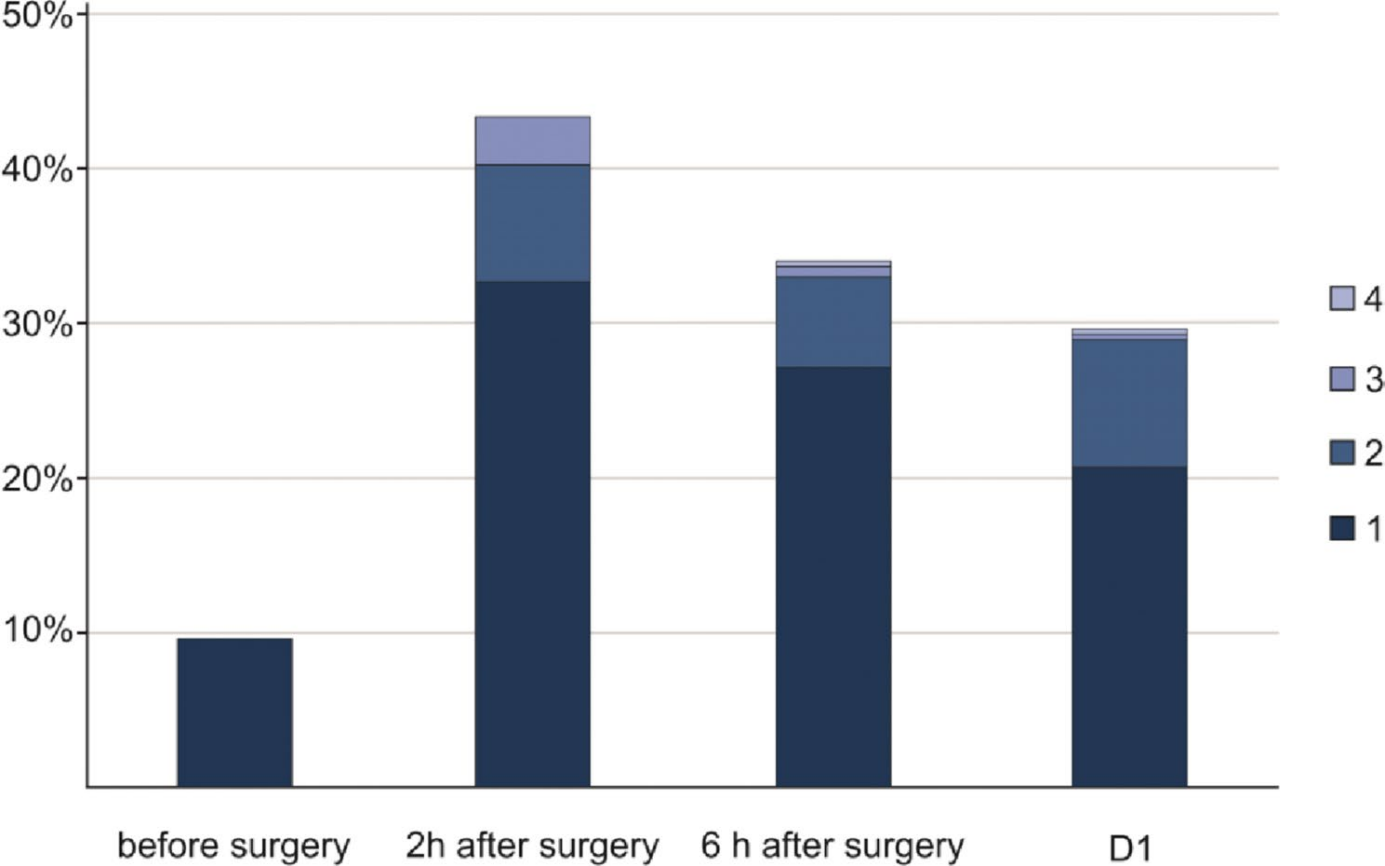
Signs of impaired tissue perfusion. Bar chart illustrating the number of patients having any sign of impaired tissue perfusion per time point. The colors indicate the number of simultaneously present signs of impaired tissue perfusion. *D1 – postoperative day 1*

171 patients (59%) had signs of impaired tissue perfusion at any postoperative time point (Figs. 1 and 2; Table 2). Specifically, capillary refill time was prolonged in 19 patients (7%), mottling score was elevated in 43 patients (15%), lactate levels were elevated in 73 patients (25%), and central venous oxygen saturation was low in 119 patients (41%). One hundred twenty-six patients (43%) had impaired tissue perfusion 2 h after the end of surgery, 99 patients (34%) six h after surgery, and 86 patients (30%) on the first postoperative day (Supplementary Fig. 1). At each time point, most patients had only a single sign of impaired tissue perfusion, i.e., 95 patients (75%) 2 h after surgery, 79 patients (80%) 6 h after surgery, and 60 patients (70%) on the first postoperative day (Fig. 2).

**Table 2:** Perioperative tissue perfusion markers

	Before surgery	2 h after surgery	6 h after surgery	Postoperative day 1
Lactate, mmol/L	0.7 (0.6 to 0.9)	1.1 (0.8 to 1.6)	1.1 (0.7 to 1.6)	1.1 (0.7 to 1.5)
Central venous oxygen saturation, %	82 (76 to 86)	70 (65 to 75)	70 (65 to 75)	70 (64 to 74)
Mottling score, au	0 (0 to 0)	0 (0 to 0)	0 (0 to 0)	0 (0 to 0)
Capillary refill time, s	1.0 (0.7 to 1.4)	1.1 (1.0 to 1.8)	1.0 (1.0 to 1.5)	1.0 (0.9 to 1.5)

Data are presented as median (25^th^ to 75^th^ percentile)

There were no meaningful differences in heart rate, blood pressure, or norepinephrine infusion rates between patients with and without signs of impaired tissue perfusion at any measurement point (Supplementary Table 1).

Patients with compared to without any postoperative sign of impaired tissue perfusion had longer intensive care unit length of stays (2 (2 to 4) days vs. 2 (2 to 3) days; *P* = 0.012), but there was no meaningful difference in maximum postoperative SOFA score, hospital length of stay, or 28-day mortality (Table 3).

**Table 3:** Comparison of patients with and without signs of impaired tissue perfusion

		Any sign of impaired tissue perfusion (*n* = 171)	No sign of impaired tissue perfusion (*n* = 120)	*P*-value
Age, years		66 (58 to 73)	62 (52 to 70)	0.017^1^
ASA physical status class, n	I	4 (2%)	6 (5%)	0.585^2^
	II	63 (37%)	47 (39%)	
	III	100 (58%)	65 (54.2%)	
	IV	4 (2%)	2 (2%)	
Duration of surgery, min		285 (208 to 370)	283 (192 to 345)	0.518^1^
Epidural catheter, n		140 (83%)	93 (78%)	0.340^3^
Intraoperative crystalloids, ml		3700 (2189 to 5173)	3368 (2125 to 4425)	0.076^1^
Intraoperative colloids, ml		240 (0 to 1000)	500 (0 to 550)	0.464^1^
Maximum postoperative SOFA score, au		3 (1 to 4)	2 (1 to 4)	0.116^1^
Maximum postoperative SOFA score ≥ 5, n		36 (21%)	18 (15%)	0.248^2^
Intensive care unit length of stay, days		2 (2 to 4)	2 (2 to 3)	0.012^1^
Hospital length of stay, days		14 (10 to 21)	13 (9 to 20)	0.528^1^
28-day mortality*, n		7/169 (4%)	3/118 (3%)	0.689^2^

Data are presented as number (percentage) or median (25^th^ to 75^th^ percentile). ^1^Kruskal-Wallis rank sum test; ^2^Pearson’s Chi-squared test; ^3^Pearson’s Chi-squared test with Yates’ continuity correction; *ASA – American Society of Anesthesiologists; SOFA – Sequential Organ Failure Assessment* *Due to loss to follow-up, 28-day mortality was assessed in only 287/291 patients

The highest postoperative lactate was weakly to moderately associated with the maximum postoperative SOFA score, intensive care unit length of stay, and hospital length of stay. The highest postoperative lactate was also higher in patients who died within 28 days compared to patients who lived. The highest postoperative mottling score was weakly associated with intensive care unit length of stay and higher in patients who died within 28 days compared to patients who lived. The longest postoperative capillary refill time was weakly associated with hospital length of stay and higher in patients who died within 28 days compared to patients who lived. There was no meaningful association between the lowest postoperative central venous oxygen saturation and the investigated outcomes (Table 4).

**Table 4:** Associations between postoperative signs of impaired tissue perfusion and postoperative outcomes

		Spearman’s ρ	95%-confidence interval	*P*-Value
*Maximum SOFA score, au*				
	Lactate, mmol/L	0.31	0.20, 0.42	< 0.001
	S_cv_O_2_, %	−0.03	−0.14, 0.09	0.659
	Mottling score, au	0.09	−0.03, 0.21	0.134
	Capillary refill time, sec	0.07	−0.01, 0.22	0.071
*Intensive care unit length of stay*,* days*				
	Lactate, mmol/L	0.13	0.02, 0.24	0.032
	S_cv_O_2_, %	−0.08	−0.20, 0.05	0.207
	Mottling score, au	0.14	0.01, 0.27	0.016
	Capillary refill time, sec	0.11	−0.02, 0.24	0.062
*Hospital length of stay*,* days*				
	Lactate, mmol/L	0.32	0.21, 0.43	< 0.001
	S_cv_O_2_, %	−0.04	−0.17, 0.07	0.492
	Mottling score, au	0.04	−0.08, 0.14	0.521
	Capillary refill time, sec	0.14	0.02, 0.26	0.015
		**Alive (n = 277)**	**Dead (n = 10)**	**P-Value**
*28-day mortality*, n*				
	Lactate, mmol/L	1.3 (0.9 to 2.0)	1.6 (1.3 to 2.3)	< 0.001
	S_cv_O_2_, %	66 (60 to 71)	54 (59 to 67)	0.336
	Mottling score, au	0 (0 to 0)	0 (0 to 0)	0.024
	Capillary refill time, sec	1.4 (1.0 to 2.0)	2.1 (1.3 to 2.8)	0.008

Data on 28-day mortality are presented as median (25^th^ to 75^th^ percentile). We used the highest postoperative lactate, mottling score, and capillary refill time and the lowest postoperative S_cv_O_2_ for the analysis. *SOFA – Sequential Organ Failure Assessment; S*_*cv*_*O*_*2*_ *– central venous oxygen saturation* *Due to loss to follow-up, 28-day mortality was assessed in only 287/291 patients

Age was weakly associated with postoperative signs of impaired tissue perfusion (median 62 (52–70) years in patients without vs. 66 (58–73) years in patients with signs of impaired tissue perfusion; *P* = 0.017), whereas no relevant association was found with the American Society of Anesthesiologists (ASA) physical status, surgery duration, intraoperative fluids, or the use of an epidural catheter (Table 3).

For the sensitivity analysis restricted to patients who developed signs of ‘new-onset impaired tissue perfusion’, we excluded 28 patients (10% of the overall cohort) who had signs of impaired tissue perfusion before surgery, and thus included 263 patients in the analysis.

One hundred fifty-two of these 263 patients (58%) developed signs of new-onset impaired tissue perfusion at any postoperative time point. Specifically, capillary refill time was prolonged in 18 patients (7%), mottling score was elevated in 38 patients (14%), lactate levels were elevated in 60 patients (23%), and central venous oxygen saturation was low in 109 patients (41%) (Supplementary Fig. 2).

The association between having any new-onset signs of impaired tissue perfusion, the highest postoperative mottling, highest postoperative capillary refill time, highest postoperative lactate, and lowest postoperative central venous oxygen saturation with postoperative maximum SOFA score, intensive care unit length of stay, hospital length of stay, and 28-day mortality are shown in Supplementary Table 2.

Age was weakly associated with postoperative new-onset signs of impaired tissue perfusion (median 61 (52–70) years in patients without vs. 66 (58–73) years in patients with signs of impaired tissue perfusion; *P* = 0.017), whereas no relevant association was found with the ASA physical status, surgery duration, intraoperative fluids, or the use of an epidural catheter.

## Discussion

In this prospective multicenter observational study, about 60% of high-risk patients having major abdominal surgery had postoperative signs of impaired tissue perfusion. However, most patients had only one sign of impaired tissue perfusion. Having a sign of postoperative tissue impairment was associated with a slightly longer intensive care unit length of stay. These primary results were confirmed in a sensitivity analysis restricted to patients who developed signs of new-onset impaired tissue perfusion.

In our study, it was common that high-risk major abdominal surgery patients had at least one sign of impaired tissue perfusion. In contrast to previous studies that mostly focused on single microcirculation markers, we assessed four different signs of impaired tissue perfusion, i.e., lactate, central venous oxygen saturation, capillary refill time, and mottling. Interestingly, most patients had only one sign of impaired tissue perfusion. An explanation may be that the individual signs of impaired tissue perfusion reflect different aspects of tissue perfusion. While capillary refill time and mottling score assess regional tissue perfusion at the fingertip and the knee or leg [11, 14], respectively, lactate production and changes in central venous oxygen saturation reflect oxygen delivery and metabolism on a systemic level. Our finding that most patients had only one sign of impaired tissue perfusion highlights a fundamental challenge in tissue perfusion monitoring: each marker provides information about different tissue perfusion compartments and may be influenced by distinct pathophysiological and compensatory mechanisms [13, 14]. The limited concordance among individual tissue perfusion markers indicates that a multimodal strategy is required for comprehensive evaluation of tissue perfusion. Single variables may capture only isolated aspects of this physiology and are unlikely to provide sufficient clinical insight. Accordingly, assessment of the microcirculation should not be based on individual variables or single measurement techniques.

Low central venous oxygen saturation was the most commonly found marker of impaired tissue perfusion in our study. A previous study with 60 patients having high-risk surgery in three European university hospitals found that the postoperative mean central venous oxygen saturation within 12 h after surgery was independently associated with postoperative complications [15]. The central venous oxygen saturation cut-off that best discriminated between patients with and without postoperative complications was 73%, which is even higher than the threshold of 65% we used in our study. However, in our study, there was no meaningful relationship between central venous oxygen saturation and poor postoperative outcomes. Low central venous oxygen saturation may not always be a sign of impaired tissue perfusion reflecting poor perfusion and oxygen delivery or increased oxygen consumption, but can, for example, also be caused by low arterial oxygen content due to reduced respiratory function or anemia.

The clinical relevance of postoperative hyperlactatemia has been investigated in several observational studies [16–19]. In a study similar to ours, postoperative lactate was evaluated 4 h, 12 h, and 24 h after major abdominal surgery in 195 patients [20]. In this study, the highest lactate levels were observed 4 h after surgery with a median value of 1.90 (1.10–2.90) mmol/L (which is substantially higher than the 1.1 (0.8 to 1.6) mmol/L we observed at 2 h after surgery). In their study, patients with postoperative lactate values of 1.35 mmol/L or higher more frequently developed postoperative complications and had a longer hospital length of stay compared to those with normal lactate [20]. Similar results were found in other prospective [17, 18] or retrospective [21] studies in major abdominal surgery patients. In line with these previous studies, we found a weak-to-moderate association between the highest postoperative lactate and maximum SOFA score, intensive care unit length of stay, hospital length of stay, and 28-day mortality. However, perioperative hyperlactatemia can also be caused by medications, liver failure, or thiamine deficiency [22, 23].

Abnormal mottling score and capillary refill time were less common in our study – and were rarely found on the first postoperative day. This may be due to the fact that surgery is regional trauma – in contrast to systemic inflammation and sepsis, in which an abnormal capillary refill time and mottling score are associated with morbidity and mortality [24]. In line, mottling scores were slightly higher and capillary refill times were slightly longer in patients who died within 28 days in our study – with weak association with intensive care unit and hospital length of stay. Nonetheless, the overall link between impaired postoperative tissue perfusion and postoperative organ injury was weak in our study. This suggests that perioperative impaired tissue perfusion is temporary and more likely due to circulatory changes than severe systemic inflammation.

About 10% of our patients had signs of impaired tissue perfusion before surgery. Most commonly, these patients had low central venous oxygen saturation (12 of 28 patients) or mottling (10 of 28 patients). Given that these were elective surgery patients, the clinical importance of this finding remains unclear. Previous studies investigating the sublingual microcirculation in populations similar to the current one, suggested that the microcirculation is usually intact before elective surgery [25–27].

Due to the COVID-19 pandemic, recruitment for our study was terminated early with a smaller sample size than initially planned. Despite their meticulous assessment, capillary refill time and mottling score were assessed manually rather than by automated methods. Consequently, precise timing of capillary refill and determination of mottling may have varied between observers. Further, body temperature is an important determinant of peripheral perfusion that was not systematically recorded during the assessments [28]. We did not systematically assess perioperative medications, such as antibiotics or metformin, which may have influenced lactate levels. Also, we did not systematically assess the sublingual microcirculation [25, 26, 29] or intraabdominal tissue perfusion [30].

In conclusion, about 60% of high-risk patients having major abdominal surgery had postoperative signs of impaired tissue perfusion. However, most patients had only one sign of impaired tissue perfusion. Whether these signs of impaired tissue perfusion are clinically meaningful requires further investigation in larger studies.

## Supplementary Information

Supplementary Material 1

Supplementary Material 2

Supplementary Material 3

Supplementary Material 4

GSAIC Trials Group

## Data Availability

De-identified data can be made available upon submission of a written request.
