# Supplementary material for: Impaired tissue perfusion in high-risk patients having major abdominal surgery: a multicenter observational study
Source: Crit Care. 2026 Mar 11;30:162. doi: 10.1186/s13054-026-05940-y (PMC13064093; doi:10.1186/s13054-026-05940-y)
Supplement: Supplementary file 1 — Supplementary Material 1 [file 13054_2026_5940_MOESM1_ESM.pdf]

**Supplementary Table 1. Macrocirculation variables**

| | | Before surgery | 2h after surgery | 6h after surgery | Postoperative day 1 |
|---|---|---|---|---|---|
| Heart rate, bpm | Overall | 55 (49 to 65) | 78 (67 to 89) | 77 (69 to 90) | 78 (70 to 88) |
| | *signs of impaired tissue perfusion* | *57 (49 to 66)* | *77 (66 to 89)* | *78 (70 to 90)* | *78 (70 to 89)* |
| | *no signs of impaired tissue perfusion* | *55 (50 to 63)* | *78 (72 to 89)* | *75 (68 to 89)* | *79 (70 to 86)* |
| Systolic arterial pressure, mmHg | Overall | 114 (105 to 125) | 121 (109 to 136) | 118 (108 to 131) | 116 (105 to 128) |
| | *signs of impaired tissue perfusion* | *115 (108 to 127)* | *120 (110 to 135)* | *118 (108 to 130)* | *116 (103 to 128)* |
| | *no signs of impaired tissue perfusion* | *112 (101 to 121)* | *123 (109 to 136)* | *118 (109 to 135)* | *114 (107 to 125)* |
| Mean arterial pressure, mmHg | Overall | 77 (72 to 85) | 80 (71 to 89) | 76 (68 to 86) | 75 (67 to 85) |
| | *signs of impaired tissue perfusion* | *79 (73 to 86)* | *79 (70 to 89)* | *76 (68 to 86)* | *74 (66 to 84)* |
| | *no signs of impaired tissue perfusion* | *76 (69 to 84)* | *80 (72 to 90)* | *78 (69 to 87)* | *76 (72 to 86)* |
| Diastolic arterial pressure, mmHg | Overall | 58 (51 to 65) | 58 (51 to 67) | 56 (49 to 64) | 55 (48 to 62) |
| | *signs of impaired tissue perfusion* | *60 (54 to 65)* | *58 (49 to 68)* | *56 (49 to 63)* | *55 (47 to 62)* |
| | *no signs of impaired tissue perfusion* | *56 (51 to 63)* | *59 (52 to 66)* | *58 (49 to 65)* | *57 (50 to 63)* |
| Norepinephrine rate, mcg/kg/min | Overall | 0.05 (0.04 to 0.09) | 0.01 (0 to 0.05) | 0 (0 to 0.05) | 0 (0 to 0.03) |
| | *signs of impaired tissue perfusion* | *0.06 (0.04 to 0.09)* | *0.02 (0 to 0.05)* | *0.01 (0 to 0.05)* | *0 (0 to 0.03)* |
| | *no signs of impaired tissue perfusion* | *0.05 (0.04 to 0.10)* | *0 (0 to 0.04)* | *0 (0 to 0.03)* | *0 (0 to 0.03)* |

Data are presented as median ($25^{th}$ to $75^{th}$ percentile) for the overall cohort and separately for patients with and without any sign of postoperative impaired tissue perfusion.
