# Supplementary material for: Impaired tissue perfusion in high-risk patients having major abdominal surgery: a multicenter observational study
Source: Crit Care. 2026 Mar 11;30:162. doi: 10.1186/s13054-026-05940-y (PMC13064093; doi:10.1186/s13054-026-05940-y)
Supplement: Supplementary file 2 — Supplementary Material 2 [file 13054_2026_5940_MOESM2_ESM.pdf]

Supplementary Table 2. Associations between postoperative *new-onset* signs of impaired tissue perfusion and postoperative outcomes

		Any sign of *new-onset* impaired tissue perfusion (n=152)	No sign of *new-onset* impaired tissue perfusion (n=111)	P-Value
Maximum postoperative SOFA score, au		3 (1 to 4)	2 (1 to 4)	0.347
Intensive care unit length of stay, days		3 (2 to 4)	2 (2 to 3)	0.011
Hospital length of stay, days		13 (10 to 20)	13 (9 to 20)	0.794
28-day mortality*, n		6 (4%)	3 (3%)	0.843
		**Spearman's ρ**	**95%-confidence interval**	**P-Value**
Maximum SOFA score, au				
	Lactate, mmol/L	0.27	0.16, 0.39	<0.001
	$S_{cv}O_2$, %	-0.04	-0.15, 0.08	0.539
	Mottling score, au	0.04	-0.09, 0.16	0.491
	Capillary refill time, sec	0.09	-0.02, 0.21	0.144
Intensive care unit length of stay, days				
	Lactate, mmol/L	0.11	-0.02, 0.24	0.074
	$S_{cv}O_2$, %	-0.10	-0.23, 0.04	0.135
	Mottling score, au	0.12	-0.01, 0.26	0.049
	Capillary refill time, sec	0.12	0, 0.25	0.058
Hospital length of stay, days				
	Lactate, mmol/L	0.30	0.18, 0.42	<0.001
	$S_{cv}O_2$, %	-0.05	-0.18, 0.08	0.453
	Mottling score, au	0.00	-0.11, 0.12	0.968

	Capillary refill time, sec	0.11	-0.02, 0.25	0.069
		**Alive (n=250)**	**Dead (n=9)**	**P-Value**
*28-day mortality,n**				
	Lactate, mmol/L	1.3 (0.9 to 1.9)	1.6 (1.3 to 2.1)	<0.001
	$S_{cv}O_2$, %	66 (60 to 72)	64 (61 to 67)	0.556
	Mottling score, au	0 (0 to 0)	0 (0 to 0)	0.273
	Capillary refill time, sec	1.5 (1.0 to 2.0)	2.1 (1.3 to 2.8)	0.018

Data on 28-day mortality are presented as median (25th to 75th percentile). We used the highest postoperative lactate, mottling score, and capillary refill time and the lowest postoperative $S_{cv}O_2$ for the analysis. *SOFA – Sequential Organ Failure Assessment; $S_{cv}O_2$ – central venous oxygen saturation* *Due to loss to follow-up, 28-day mortality was assessed in only 259/263 patients.
