# Supplementary material for: Impaired tissue perfusion in high-risk patients having major abdominal surgery: a multicenter observational study
Source: Crit Care. 2026 Mar 11;30:162. doi: 10.1186/s13054-026-05940-y (PMC13064093; doi:10.1186/s13054-026-05940-y)
Supplement: Supplementary file 3 — Supplementary Material 3 [file 13054_2026_5940_MOESM3_ESM.pdf]

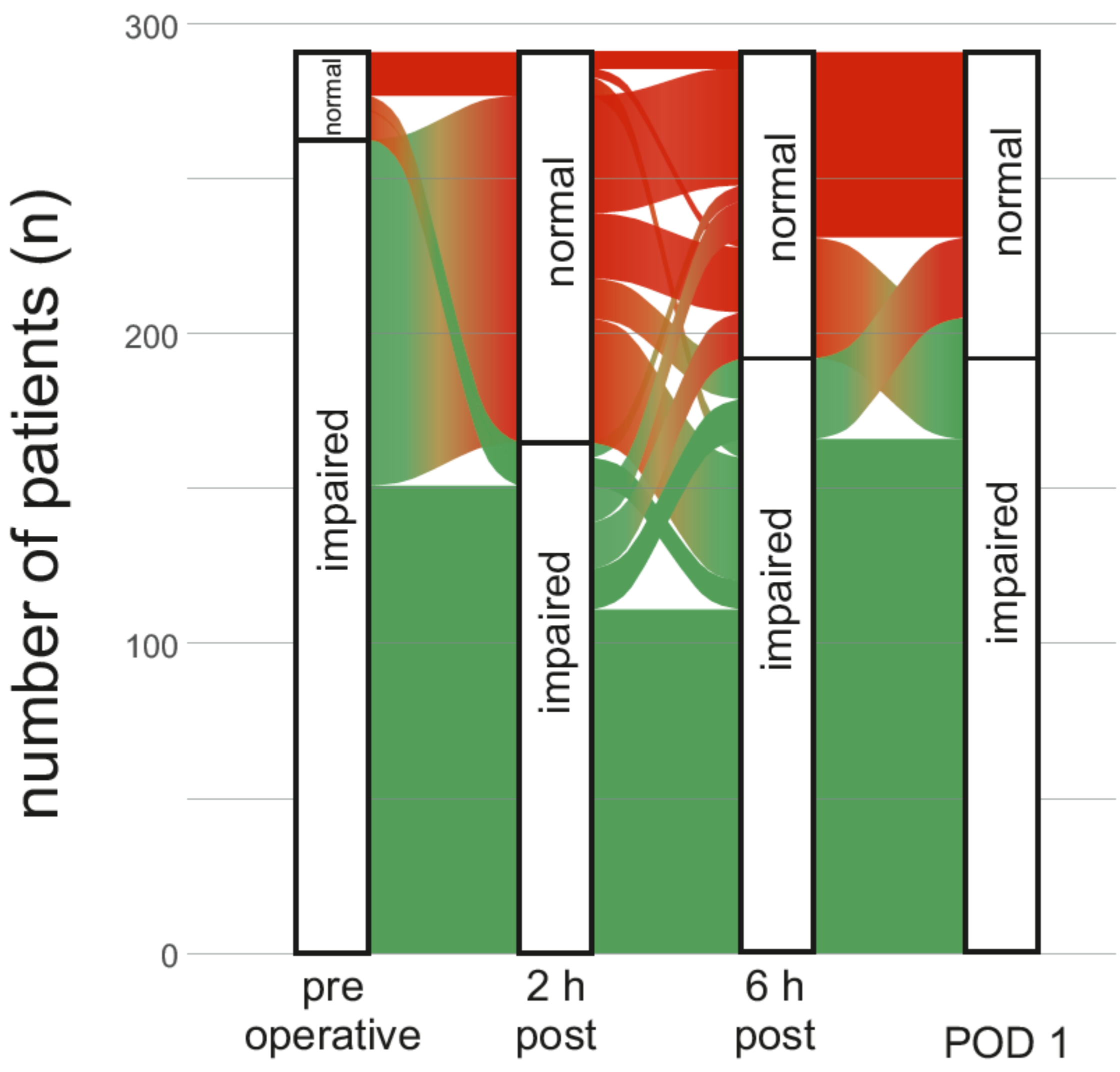

Supplementary Figure 1: Evolution of impaired tissue perfusion.

Sankey illustrating the evolution of patients having signs of impaired tissue perfusion per time point. *POD 1– postoperative day 1*
