# Supplementary material for: Impaired tissue perfusion in high-risk patients having major abdominal surgery: a multicenter observational study
Source: Crit Care. 2026 Mar 11;30:162. doi: 10.1186/s13054-026-05940-y (PMC13064093; doi:10.1186/s13054-026-05940-y)
Supplement: Supplementary file 4 — Supplementary Material 4 [file 13054_2026_5940_MOESM4_ESM.pdf]

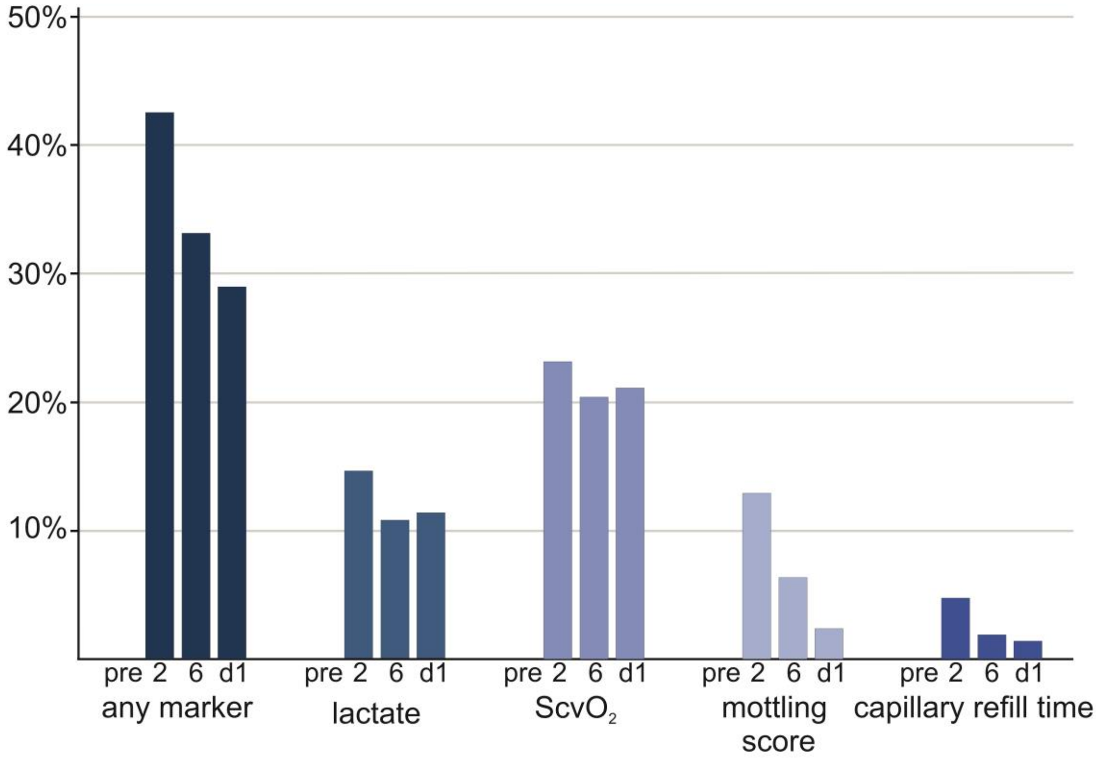

Supplementary Figure 2: Individual signs of new-onset impaired tissue perfusion.

Bar chart illustrating the number of patients having each sign of new-onset impaired tissue perfusion per time point. *ScvO2 – central venous oxygen saturation*; *D1 – postoperative day 1*
