# Supplementary material for: Impaired tissue perfusion in high-risk patients having major abdominal surgery: a multicenter observational study
Source: Crit Care. 2026 Mar 11;30:162. doi: 10.1186/s13054-026-05940-y (PMC13064093; doi:10.1186/s13054-026-05940-y)
Supplement: Supplementary file 5 — GSAIC Trials Group [file 13054_2026_5940_MOESM5_ESM.pdf]

Investigators of the *German Society of Anaesthesiology and Intensive Care (GSAIC) Trials Group:*

Alexander Fuchs	Department of Anesthesiology and Intensive Care Medicine, Faculty of Medicine, University Hospital of Cologne, University of Cologne, Cologne, Germany
Dominik Wrede	Department of Anesthesiology, Intensive Care Medicine and Pain Therapy, University Medical Center Rostock, Rostock, Germany
Lena Bork	Department of Anesthesiology, Intensive Care Medicine and Pain Therapy, University Medical Center Rostock, Rostock, Germany
Raimund Huf	Department of Anaesthesiology and Intensive Care Medicine, University Hospital Ulm, Ulm, Germany
Axel Nierhaus	Department of Intensive Care Medicine, University Medical Center Hamburg-Eppendorf, Hamburg, Germany
Karim Kouz	Department of Anesthesiology, Center of Anesthesiology and Intensive Care Medicine, University Medical Center Hamburg-Eppendorf, Hamburg, Germany
Sarah S Grotheer	Department of Anaesthesiology & Intensive Care Medicine, TUM University Hospital Rechts der Isar, Munich, Germany<br>Department of Anesthesia, Critical Care and Pain Medicine, Hessing Foundation, Augsburg, Germany
Sophie Perchermaier	Department of Anaesthesiology & Intensive Care Medicine, TUM University Hospital Rechts der Isar, Munich, Germany
Anna Scholze	Department of Anaesthesiology & Intensive Care Medicine, TUM University Hospital Rechts der Isar, Munich, Germany
